# Impact of grape seed proanthocyanidin B2 pretreatment on mitochondrial oxidative stress, endoplasmic reticulum stress, and apoptosis in renal tubular epithelial cells during *in-vitro* hypoxia-reoxygenation

**DOI:** 10.1590/acb404125

**Published:** 2025-06-06

**Authors:** Zhi-shun Wang, Qi Han, Hao Shen, Bo Shu, Cheng-cheng Ying, Guo-hao Li, Yong-lian Guo

**Affiliations:** 1 Huazhong University of Science and Technology – Tongji Medical College –The Central Hospital of Wuhan – Department of Urology – Wuhan, People’s Republic of China.; 2 Wuhan University of Science and Technology – Geriatric Hospital – Hemodialysis Center – Wuhan, People’s Republic of China.

**Keywords:** Oxidative Stress, Membrane Potential, Mitochondrial, Endoplasmic Reticulum Stress, Apoptosis

## Abstract

**Purpose:**

To investigate the impact of grape seed proanthocyanidin B2 (GSPB2) pretreatment on hypoxia-reoxygenation model of HK-2 cells *in vitro*.

**Methods:**

The experiment was divided into five groups: control group (control), GSPB2 group (GSPB2), hypoxia-reoxygenation group (HR), GSPB2 + HR group (GSPB2+HR), and GSPB2 + brusatol (BRU) + HR group (GSPB2 + BRU + HR). Flow cytometry was used to detect apoptosis of cells. Transmission electron microscopy was employed to observe ultrastructural changes of cells. Mitochondrial membrane potential (MMP) was measured. Cellular immunofluorescence was used to assess intracellular Ca^²+^ concentration. Immunofluorescence staining and Western blotting were conducted to detect expression levels of Nrf2, HO-1, glucose-regulated protein 78 (GRP78), C/EBP homologous protein (CHOP), and cleaved-caspase3.

**Results:**

Compared to HR group, GSPB2 + HR group showed significantly increased cell viability, and reduced mitochondrial damage in the cytoplasm. MMP in GSPB2 + HR group was significantly restored, and intracellular Ca^²+^ concentration was significantly decreased. The expression of Nrf2 and HO-1 proteins was significantly upregulated, while the expression of GRP78, CHOP, and cleaved-caspase3 proteins was markedly downregulated.

**Conclusion:**

GSPB2 pretreatment can alleviate oxidative stress damage, mitochondrial injury, and endoplasmic reticulum stress induced by hypoxia-reoxygenation in HK-2 cells *in vitro*. This effect may be related to the ability of GSPB2 pretreatment to activate the endogenous antioxidant system, particularly through the activation of the Nrf2/HO-1 signaling pathway.

## Introduction

Renal ischemia-reperfusion injury is one of the main causes of acute kidney injury. This type of injury can occur not only during kidney transplantation but also frequently in clinical settings involving procedures such as nephron-sparing surgeries, cardiac surgeries, septicemia, and shock from various causes^1^. The pathophysiological process of ischemia-reperfusion injury is very complex, involving multiple factors, including cellular hypoxia and energy metabolism disturbances, calcium overload, excessive production of reactive oxygen species (ROS) and oxidative stress damage, inflammatory responses and production of pro-inflammatory factors, as well as cellular necrosis and apoptosis^2^^,^ 3. Excessive production of oxygen free radicals by mitochondria can disrupt adenosine triphosphate (ATP) generation, lead to dysregulation of intracellular calcium levels, and result in the opening of the mitochondrial permeability transition pore, which subsequently causes apoptosis or necrosis^4^. Mitochondrial oxidative stress plays a crucial role in the process of ischemia-reperfusion injury. Therefore, alleviating mitochondrial oxidative stress damage and maintaining mitochondrial membrane potential are of great significance in mitigating renal ischemia-reperfusion injury.

Proanthocyanidins are natural polyphenolic compounds that are widely distributed in nature, primarily found in plants such as grapes, apples, cherries, and peanuts. Among them, grape seed proanthocyanidin B2 (a proanthocyanidin dimer) has good water solubility, is easily absorbed, and possesses strong antioxidant activity^5^. Previous studies have shown that grape seed proanthocyanidin B2 exhibits excellent antioxidant, anti-inflammatory, and anti-apoptotic properties^6^^,^ 7.

This study aimed to construct a hypoxia-reoxygenation model in renal tubular epithelial cells and, based on this model, further explore the effects of grape seed proanthocyanidin B2 pretreatment on the human renal tubular epithelial cell hypoxia-reoxygenation model and the potential underlying mechanisms.

## Methods

### Drugs

Grape seed proanthocyanidin B2 and brusatol were both purchased from Shanghai Aladdin Biochemical Technology Co., Ltd.

### Preparation of HK-2 cell hypoxia-reoxygenation model

According to the experimental requirements, HK-2 cells were seeded into culture dishes and incubated in a 5% CO_2_ incubator. The dishes were removed from the incubator, the original medium was discarded, and the cells were washed three times with pre-warmed Earle’s balanced salt solution (EBSS without glucose). The dishes were then transferred to a custom-made hypoxia chamber with conditions set to 37°C and a gas mixture of 95% N_2_ and 5% CO_2_. After 12 hours of hypoxia, the cells were removed from the hypoxia chamber, the glucose-free EBSS was discarded, and pre-warmed standard culture medium was added. The cells were then incubated under normal conditions (37°C, 5% CO_2_) for an additional 4 hours, thereby establishing the HK-2 cell hypoxia-reoxygenation model.

### Experimental groups and treatment

The *in-vitro* study was divided into five experimental groups, with three cell culture plates used for each group when testing different indicators:

- Control group (control): HK-2 cells were cultured under normal conditions in the incubator;
- Grape seed proanthocyanidin B2 group (GSPB2): HK-2 cells were cultured under normal conditions in the incubator, with grape seed proanthocyanidin B2 added to the culture medium;
- Hypoxia-reoxygenation group (HR): HK-2 cells were cultured under glucose deprivation and hypoxic conditions in the incubator for 12 hours, followed by reoxygenation and restoration to normal culture conditions for an additional 4 hours;
- Grape seed proanthocyanidin B2 + hypoxia-reoxygenation group (GSPB2 + HR): HK-2 cells are exposed to glucose deprivation and hypoxic conditions for 12 hours, then grape seed proanthocyanidin B2 was added before reoxygenation. The cells were then reoxygenated and cultured under normal conditions for an additional 4 hours.
- Grape seed proanthocyanidin B2 + brusatol (Nrf2 Inhibitor) + hypoxia-reoxygenation group (GSPB2 + BRU + HR): HK-2 cells were subjected to glucose deprivation and hypoxic conditions for 12 hours. Before reoxygenation, grape seed proanthocyanidin B2 and the Nrf2 inhibitor brusatol (BRU) were added. The cells were then reoxygenated and cultured under normal conditions for an additional 4 hours.

### Flow cytometry analysis

Cells in the logarithmic growth phase and in good condition were seeded into 96-well plates at the density of 5 × 10^3^ cells per well and incubated overnight at 37°C. The cells were then treated according to the following experimental groups:

- Control: HK-2 cells were cultured under normal conditions in the incubator;
- GSPB2: HK-2 cells were cultured under normal conditions in the incubator, with grape seed proanthocyanidin B2 (10 µmol/L) added to the culture medium;
- HR: HK-2 cells were cultured under glucose deprivation and hypoxic conditions in the incubator for 12 hours, followed by reoxygenation for 4 hours and restoration to normal culture conditions;
- GSPB2 + HR: HK-2 cells were treated with grape seed proanthocyanidin B2 (10 µmol/L) and then subjected to glucose deprivation and hypoxic conditions for 12 hours, followed by reoxygenation for 4 hours and restoration to normal culture conditions;
- GSPB2 + BRU + HR: HK-2 cells were treated with grape seed proanthocyanidin B2 (10 µmol/L) and the Nrf2 inhibitor berbamine (BRU) (4 µg/mL).

After 12 hours of glucose deprivation and hypoxia, the cells were reoxygenated for 4 hours and then returned to normal culture conditions. After the required incubation time, the cells were collected and centrifuged at 1,000 rpm for 5 minutes. The supernatant was discarded, and the cells were resuspended in phosphate buffered saline (PBS). The cells were then washed twice with PBS, centrifuging each time at 1,000 rpm for 5 minutes.

The procedure was followed according to the instructions provided with the FITC/PI apoptosis detection kit:

- Add 500 μL of binding buffer to resuspend the cells;
- Add 5 μL of Annexin V-FITC and mix thoroughly, then add 5 μL of PI and mix well;
- Incubate the cells at room temperature in the dark for 5-15 minutes. A negative control must be set up at the same time, using normal cells without Annexin V-FITC and PI;
- Analyze the samples using a flow cytometer. Analyze the apoptosis rate using FlowJo 8.7.1 software.

### Transmission electron microscopy observation

The procedure was:

- Collect the HK-2 cells and centrifuge them;
- Add 2.5% glutaraldehyde solution for fixation at 4°C for 2 hours;
- Wash the cells three times with 0.1 mol/L PBS, 15 minutes per wash;
- Fix the cells with 1% osmium tetroxide for 1 to 1.5 hour, and then wash them three times with 0.1 mol/L PBS, 15 minutes per wash. The cells were then fixed with 1% osmium tetroxide, followed by dehydration through an ethanol gradient and embedding in resin;
- Double stain the samples with 2% uranyl acetate and lead citrate;
- Observe the samples and capture images using a transmission electron microscope (JEM-1200EX).

### Detection of mitochondrial membrane potential

The procedure was:

- Use the JC-1 mitochondrial membrane potential analysis kit to detect the mitochondrial membrane potential levels in HK-2 cells from each group. After successfully establishing the model, place the cells in a tri-gas incubator;
- According to the instructions, add JC-1 solution to the cells and incubate them at 37°C for 30 minutes;
- Wash the cells three times with PBS;
- Observe and capture images of the cells under a fluorescence microscope.

When the mitochondrial membrane potential is high or normal, JC-1 aggregates in the mitochondrial matrix and forms polymers, producing red fluorescence. Conversely, when the mitochondrial membrane potential is reduced, JC-1 remains as monomers and cannot aggregate in the mitochondrial matrix, emitting green fluorescence.

### Detection of intracellular Ca^2+^ concentration

The procedure was:

- Use the fluorescence probe method to detect intracellular Ca^2+^ concentrations in HK-2 cells from each group, with Fura-2 AM (from Beyotime) as the fluorescent dye;
- After successfully establishing the model and placing the cells in a tri-gas incubator, add the Fura-2 AM working solution to the cells;
- Incubate the cells at 37°C for 30 minutes;
- Wash the cells three times with 0.1 mol/L PBS;
- Observe and capture images of the cells under a fluorescence microscope.
  Cell immunofluorescence staining

The procedure was:

- Rinse the coverslips with cells in the culture plates three times with PBS, 3 minutes each time;
- Fix the coverslips with 4% paraformaldehyde for 15 minutes, then wash them with 0.1 mol/L PBS three times, 3 minutes each time;
- Permeabilize the cells with 0.5% Triton X-100 (prepared in PBS) at room temperature for 5 minutes;
- Wash the coverslips with 0.1 mol/L PBS three times, 3 minutes each time;
- Blot the PBS dry with absorbent paper, then add normal goat serum to the coverslips and incubate at room temperature for 30 minutes to block non-specific binding;
- Remove the blocking solution with absorbent paper without washing. Add an appropriate amount of diluted primary antibody to each coverslip and place the coverslips in a humidified chamber. Incubate at 4°C overnight;
- Add fluorescent secondary antibody: wash the coverslips with phosphate-buffered saline with Tween (PBST) three times, 3 minutes each time. Then, add the appropriately diluted fluorescent secondary antibody and incubate it in a humidified chamber at 37°C for 1 hour. Wash the coverslips with PBST three times, 3 minutes each time;
- Nuclear staining: Add DAPI and incubate it in the dark for 5 minutes to stain the nuclei. Wash the coverslips with PBST four times, 5 minutes each time, to remove excess DAPI. Finally, blot the coverslips dry with absorbent paper, then mount them using an antifade mounting medium that contains anti-fluorescence quenching agents. Observe and capture images under a fluorescence microscope.

### Western blot analysis

The procedure was:

- Seed HK-2 cells in the logarithmic growth phase into culture flasks;
- Place the flasks in a tri-gas incubator and, after successful modeling, collect the HK-2 cells from each group and extract the cellular proteins;
- Separate the proteins using SDS-PAGE gel electrophoresis, then transfer the proteins onto a polyvinylidene difluoride (PVDF) membrane;
- Soak the PVDF membrane in 5% non-fat milk and block it at room temperature for 2 hours;
- Incubate the PVDF membrane overnight at 4°C with the following primary antibodies, each diluted 1:1,000: Nrf2, HO-1, glucose-regulated protein 78 (GRP78), C/EBP homologous protein (CHOP), and cleaved-caspase3;
- After incubation, wash the PVDF membrane with Tris-buffered saline with Tween-20 (TBST) three times, each for 10 minutes. Then, add the corresponding HRP-conjugated secondary antibody and incubate it at room temperature on a shaker for 2 hours;
- Wash the membrane again with TBST three times, then perform enhanced chemiluminescence (ECL) detection and imaging to visualize the protein bands.

### Statistical analysis

All data were statistically analyzed using Statistical Package for the Social Sciences 24.0 software. The measurement data were expressed as mean ± standard deviation (mean ± SD). Differences between two groups were assessed using the t-test. A *p*-value less than 0.05 (*p* < 0.05) was considered statistically significant.

## Results

### Pretreatment with grape seed proanthocyanidin B2 significantly reduces apoptosis induced by hypoxia-reoxygenation in HK-2 cells

**Figure 1:**
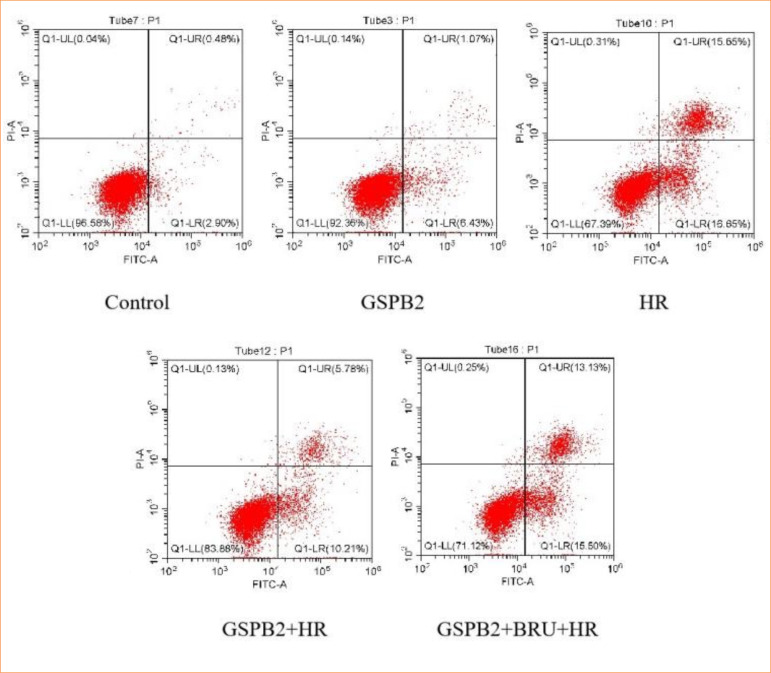
Flow cytometry detection of apoptosis in HK-2 cells across different experimental groups.

GSPB2: grape seed proanthocyanidin B2; HR: hypoxia-reoxygenation; BRU: brusatol. Source: Elaborated by the authors.

**Figure 2:**
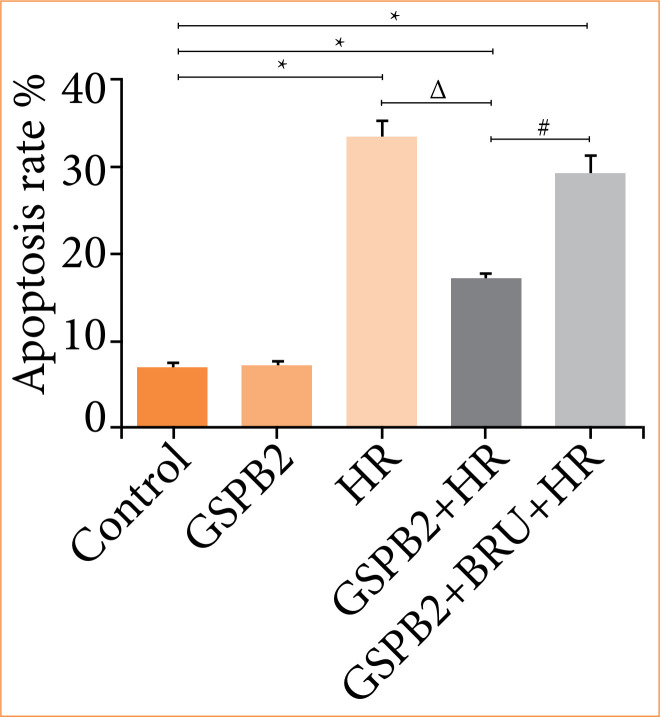
Flow cytometry detection of apoptosis rates in HK-2 cells across different experimental groups.

GSPB2: grape seed proanthocyanidin B2; HR: hypoxia-reoxygenation; BRU: brusatol; *p < 0.05 versus control group, Δ*p* < 0.05 versus HR group, #*p* < 0.05 *versus* GSPB2 + HR group. Source: Elaborated by the authors.

We directly assessed the apoptosis rate of cells in each group using flow cytometry. Flow cytometry results showed that, compared to the control group, the apoptosis rates in the HR group, GSPB2 + HR group, and GSPB2 + BRU + HR group were all significantly increased, with statistically significant differences (*p* < 0.05). Among these, the HR group had the highest apoptosis rate. Compared to the HR group, the apoptosis rate in the GSPB2 + HR group was significantly reduced, with a statistically significant difference (*p* < 0.05). Conversely, compared to the GSPB2 + HR group, the apoptosis rate in the GSPB2 + BRU + HR group was significantly increased, with a statistically significant difference (*p* < 0.05) (Figs. 1 and 2).

### Grape seed proanthocyanidin B2 pretreatment significantly alleviates hypoxia-reoxygenation-induced mitochondrial damage in HK-2 cells

**Figure 3:**
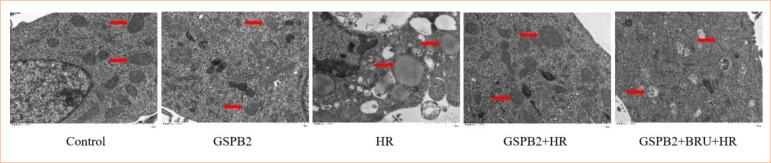
Transmission electron microscopy was used to observe the mitochondrial ultrastructure of HK-2 cells in each group (magnification ×12,000). The structure indicated by the red arrow is the mitochondrion of HK-2 cells in each group.

GSPB2: grape seed proanthocyanidin B2; HR: hypoxia-reoxygenation; BRU: brusatol. Source: Elaborated by the authors.

Transmission electron microscopy observations indicated that, compared to the control group, the HR group exhibited severe mitochondrial damage in HK-2 cells. In the HR group, the mitochondria were markedly swollen and rounded, with mitochondrial cristae fragmented or even absent, and some mitochondria displayed vacuolar degeneration. Compared to the HR group, the GSPB2 + HR group showed a significant reduction in mitochondrial morphological damage in HK-2 cells, including swelling, cristae fragmentation, and vacuolar degeneration. However, in the GSPB2 + BRU + HR group, the addition of BRU led to a significant reduction in the protective effect of GSPB2 on mitochondria. See Fig. 3.

### Grape seed proanthocyanidin B2 pretreatment helps restore the mitochondrial membrane potential in HK-2 cells induced by hypoxia-reoxygenation

**Figure 4:**
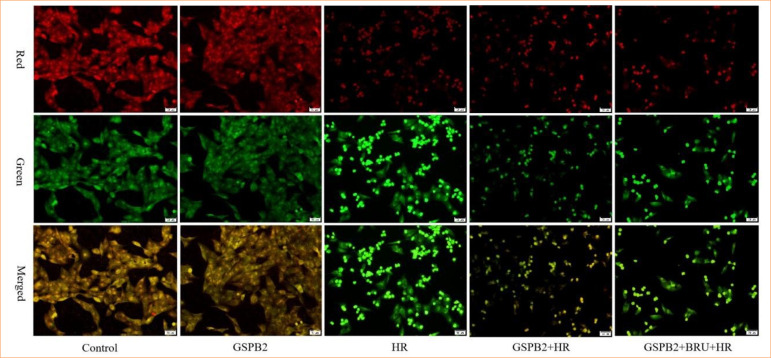
JC-1 staining was used to detect the mitochondrial membrane potential of HK-2 cells in each group (magnification ×200).

GSPB2: grape seed proanthocyanidin B2; HR: hypoxia-reoxygenation; BRU: brusatol. Source: Elaborated by the authors.

We indirectly assessed the mitochondrial membrane potential of HK-2 cells in each group using JC-1 staining. The results of mitochondrial membrane potential detection by JC-1 showed that the red fluorescence of HK-2 cells in the control group and GSPB2 group was stronger, while the green fluorescence was weaker. However, the green fluorescence of HK-2 cells in the HR group was significantly enhanced, while the red fluorescence was significantly weakened. The results indicated that hypoxia-reoxygenation caused a significant decrease in the mitochondrial membrane potential of HK-2 cells, as well as mitochondrial damage. In the GSPB2 + HR group, pretreatment with GSPB2 led to a partial recovery of the red fluorescence within the mitochondria and an increase in mitochondrial membrane potential. This suggested that GSPB2 pretreatment has a protective effect on maintaining mitochondrial membrane potential stability. However, when the Nrf2 inhibitor BRU was added, the protective effect of GSPB2 pretreatment on mitochondrial membrane potential was inhibited. See Fig. 4.

### Grape seed proanthocyanidin B2 pretreatment could significantly reduce hypoxia-reoxygenation induced Ca^2+^ overload in HK-2 cells

**Figure 5:**
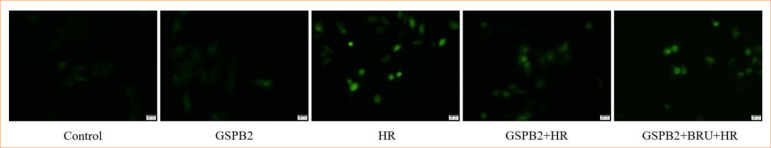
The fluorescent probe Fura-2 AM was used to detect the intracellular calcium ion concentration in HK-2 cells in each group (magnification ×400).

GSPB2: grape seed proanthocyanidin B2; HR: hypoxia-reoxygenation; BRU: brusatol. Source: Elaborated by the authors.

We indirectly assessed the intracellular calcium ion concentration of HK-2 cells in each group using the fluorescent probe Fura-2 AM. The results showed that the green fluorescence intensity of HK-2 cells in the control group was extremely weak, while the green fluorescence of HK-2 cells in the HR group was significantly enhanced, indicating that the hypoxia-reoxygenation intervention led to the calcium overload of HK-2 cells. However, in the GSPB2 + HR group, the green fluorescence intensity of HK-2 cells was significantly weakened, indicating that grape seed proanthocyanidin B2 pretreatment could significantly alleviate the calcium overload of HK-2 cells induced by hypoxia-reoxygenation. However, when the Nrf2 inhibitor BRU was added, the protective effect of GSPB2 pretreatment against hypoxia-reoxygenation-induced Ca2+ overload in HK-2 cells was partially inhibited. See Fig. 5.

### Grape seed proanthocyanidin B2 pretreatment can significantly enhanced the expression of antioxidant proteins Nrf-2 and HO-1

**Figure 6:**
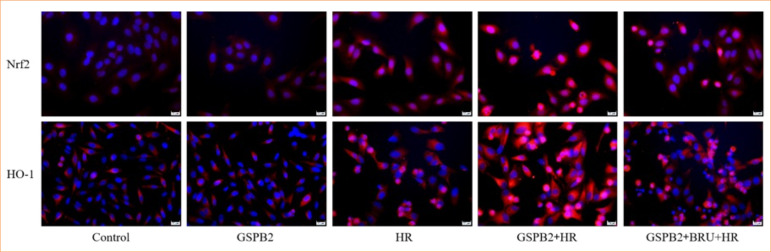
Immunofluorescence staining was used to detect the expression of the antioxidant proteins Nrf2 and HO-1 in HK-2 cells in each group.

GSPB2: grape seed proanthocyanidin B2; HR: hypoxia-reoxygenation; BRU: brusatol. Source: Elaborated by the authors.

**Figure 7:**
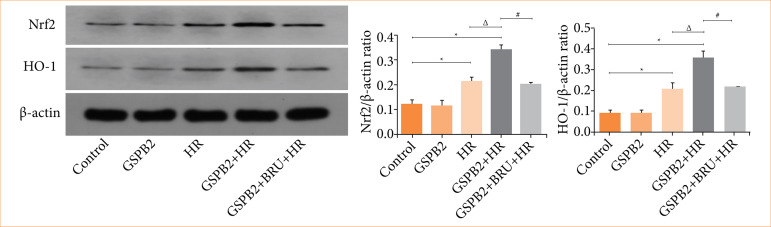
Western blotting was used to detect the expression of the antioxidant proteins Nrf2 and HO-1 in HK-2 cells in each group.

GSPB2: grape seed proanthocyanidin B2; HR: hypoxia-reoxygenation; BRU: brusatol; *p < 0.05 versus control group, Δ*p* < 0.05 versus HR group, #*p* < 0.05 *versus* GSPB2 + HR group. Source: Elaborated by the authors.

We directly detected the expression of the antioxidant proteins Nrf-2 and HO-1 in HK-2 cells of each group using immunofluorescence staining and Western blotting. The results of immunofluorescence staining and Western blotting showed that the expression levels of the antioxidant proteins Nrf2 and HO-1 were low in both the control group and GSPB2 group. In the HR group, the protein expression levels of Nrf2 and HO-1 were upregulated to a certain extent due to the induction by hypoxia-reoxygenation. Moreover, compared with HR group, the protein expression levels of Nrf2 and HO-1 in HK-2 cells in the GSPB2 + HR group were significantly up-regulated, and the difference was statistically significant (*p* < 0.05). However, in the GSPB2 + BRU + HR group, the addition of the Nrf2 inhibitor BRU resulted in a partial suppression of Nrf2 and HO-1 protein expression. See Figs. 6 and 7.

### Grape seed proanthocyanidin B2 pretreatment can significantly inhibit the expression of endoplasmic reticulum stress-related proteins GRP78, CHOP, and the apoptosis protein cleaved-caspase3

**Figure 8:**
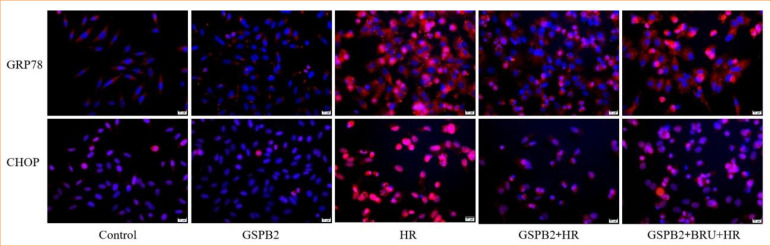
Immunofluorescence staining was used to detect the expression of GRP78 and CHOP proteins in HK-2 cells in each group.

GSPB2: grape seed proanthocyanidin B2; HR: hypoxia-reoxygenation; BRU: brusatol. Source: Elaborated by the authors.

**Figure 9:**
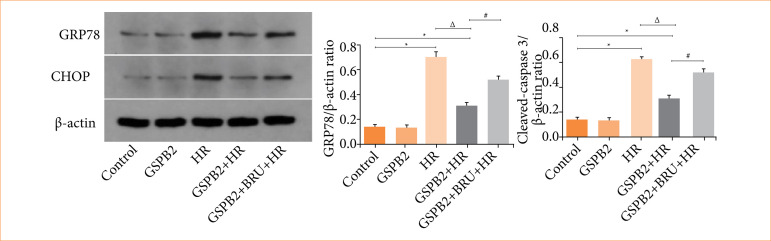
Western blotting were used to detect the expression of GRP78 and CHOP proteins in HK-2 cells in each group.

GSPB2: grape seed proanthocyanidin B2; HR: hypoxia-reoxygenation; BRU: brusatol; *p < 0.05 versus control group, Δp < 0.05 versus HR group, #p < 0.05 versus GSPB2 + HR group. Source: Elaborated by the authors.

**Figure 10:**
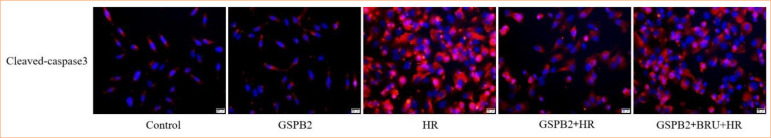
Immunofluorescence staining was used to detect the protein expression of cleaved-caspase3 in HK-2 cells in each group.

GSPB2: grape seed proanthocyanidin B2; HR: hypoxia-reoxygenation; BRU: brusatol. Source: Elaborated by the authors.

**Figure 11:**
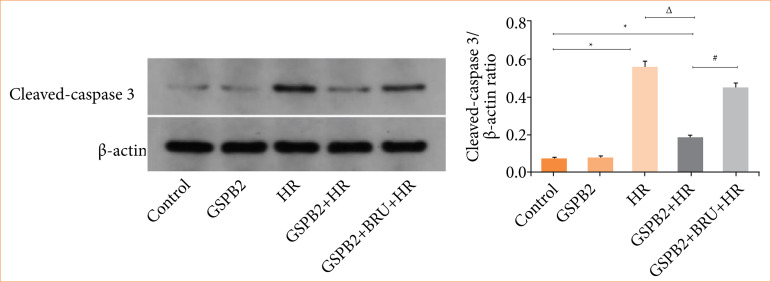
Western blotting was used to detect the protein expression of cleaved-caspase3 in HK-2 cells in each group.

GSPB2: grape seed proanthocyanidin B2; HR: hypoxia-reoxygenation; BRU: brusatol; *p < 0.05 versus control group, Δp < 0.05 versus HR group, #p < 0.05 versus GSPB2 + HR group. Source: Elaborated by the authors.

We directly detected the protein expression levels of GRP78, CHOP, and cleaved-caspase3 in HK-2 cells of each group using immunofluorescence staining and Western blotting. The results of cellular immunofluorescence staining and Western blotting showed that the expression levels of the endoplasmic reticulum stress-related proteins GRP78 and CHOP, as well as the apoptosis protein cleaved-caspase3, were low in both the control group and the GSPB2 group. Due to the induction by hypoxia-reoxygenation, the expression levels of the endoplasmic reticulum stress-related proteins GRP78 and CHOP, as well as the apoptosis protein cleaved-caspase3, were significantly upregulated in the HR group of HK-2 cells. In comparison to the HR group, the GSPB2 + HR group showed a significant downregulation in the expression levels of the endoplasmic reticulum stress-related proteins GRP78 and CHOP, as well as the apoptosis protein cleaved-caspase3. These differences were statistically significant (*p* < 0.05). However, in the GSPB2 + BRU + HR group, due to the combined intervention of GSPB2 and the Nrf2 inhibitor BRU, the expression levels of the endoplasmic reticulum stress-related proteins GRP78 and CHOP, as well as the apoptosis protein cleaved-caspase3, were elevated to a certain extent in HK-2 cells. See Figs. 8, 9, 10, 11.

## Discussion

Kidney ischemia-reperfusion injury often occurs during renal transplantation, nephron-sparing surgery, acute myocardial infarction, shock, and sepsis. It is one of the major causes of acute kidney injury. The pathological process of reperfusion injury is quite complex. It is currently believed that ischemia-reperfusion injury involves multiple pathological changes, including impaired cellular energy metabolism, excessive ROS formation, mitochondrial oxidative stress damage, endoplasmic reticulum stress, calcium overload, complement activation, inflammatory responses, and both necrosis and apoptosis^8^^,^ 9. It is a complex cascade reaction process in which multiple factors and systems participate collectively. Currently, in clinical practice, strategies such as minimizing renal ischemia time, promptly restoring renal blood supply, renal cryopreservation, and mechanical perfusion are primarily used to mitigate renal ischemia-reperfusion injury^10^. At present, numerous interventions have been studied to alleviate renal ischemia-reperfusion injury, including stem cell transplantation, mitochondrial transplantation, and novel drug therapies^11^^–^ 13.

Previous studies have shown that grape seed proanthocyanidins can alleviate endoplasmic reticulum stress and stress-induced apoptosis during ischemic stroke^14^. Grape seed proanthocyanidins mitigate H_2_O_2_-induced mitochondrial dysfunction and apoptosis by activating SIRT1 in embryonic kidney cells^15^. Grape seed proanthocyanidins can also improve cadmium-induced renal injury and oxidative stress in experimental rats by upregulating nuclear factor erythroid 2-related factor 2 (Nrf2) and antioxidant response elements^16^.

Among the many effective components of grape seed proanthocyanidins, the proanthocyanidin dimers exhibit the highest antioxidant activity and are well absorbed in the body. Studies have shown that proanthocyanidin-B2 can mitigate cadmium-induced uterine toxicity in rats by upregulating antioxidant enzymes glutathione peroxidase (GSH-Px) and superoxide dismutase (SOD), and by decreasing levels of tumor necrosis factor-α, interleukin-1β, and interleukin-6^17^. Similarly, recent studies have shown that proanthocyanidin-B2 can reduce oxidative stress damage induced by oxidized low-density lipoprotein (LDL) in human umbilical vein endothelial cells by promoting the recovery of mitochondrial membrane potential and decreasing levels of ROS and malondialdehyde (MDA)^18^.

In this study, we found that pretreatment with grape seed proanthocyanidin B2 significantly reduced apoptosis of renal tubular epithelial cells induced by hypoxia-reoxygenation, as demonstrated by flow cytometry. Additionally, transmission electron microscopy observations revealed that pretreatment with grape seed proanthocyanidin-B2 significantly alleviated mitochondrial damage in renal tubular epithelial cells. Moreover, JC-1 assay results indicated that grape seed proanthocyanidin-B2 pretreatment helped restore mitochondrial membrane potential in HK-2 cells.

Currently, it is believed that during the ischemic and hypoxic stages, cells primarily rely on anaerobic metabolism to produce ATP, leading to insufficient intracellular ATP production. The lack of ATP impairs the activity of energy-dependent sodium-potassium pumps on the cell membrane, causing abnormal increases in intracellular Na^+^ retention and concentration, and a decrease in intracellular antioxidant production. To lower intracellular Na+ levels, the Na^+^-Ca^2+^ exchanger on the cell membrane is compensatorily involved in regulation, leading to an abnormal increase in intracellular Ca^2+^ concentration to maintain the cell membrane potential19. At the same time, due to the lack of ATP, the calcium pumps on the cell membrane become dysfunctional and cannot effectively remove excess Ca^2+^ from the cell. Additionally, malfunction of the calcium pumps in the endoplasmic reticulum further obstructs the reuptake of Ca^2+^. The accumulation of multiple unfavorable factors eventually leads to intracellular Ca^2+^ overload. This overload can cause cell damage through various mechanisms, including increased intracellular ROS production, interference with mitochondrial function, disruption of cell membrane structure, and induction of apoptosis^19^. Additionally, the abnormal rise in intracellular Na^+^ concentration can reduce the activity of sodium–hydrogen exchangers, leading to the accumulation of intracellular H^+^ and a decrease in pH. This results in metabolic acidosis, which impairs the activity of cytoplasmic enzymes and disrupts protein synthesis. However, excessive intracellular Ca^2+^, Na^+^, and H^+^ create a hypertonic environment, causing water to enter the cell. This leads to cell swelling and can ultimately induce apoptosis or necrosis. In this study, pretreatment with grape seed proanthocyanidin B2 significantly reduced Ca^2+^ overload in HK-2 cells caused by hypoxia-reoxygenation. This effect may be due to its role in alleviating oxidative stress, maintaining mitochondrial membrane potential, supporting ATP production, and preserving the function of ion pumps on the cell membrane.

Nrf2 is a crucial redox-sensitive transcription factor. It directly counters the effects of free radicals and regulates cellular antioxidant activity by controlling the transcription of various detoxification genes^20^. Under normal conditions, Nrf2 is sequestered in the cytoplasm by Keap1, which binds to Nrf2 via the Cul3 ubiquitin ligase complex containing E3. This interaction targets Nrf2 for degradation by proteasomes, thereby maintaining its low levels. Upon exposure to oxidative stress, Keap1 acts as a sensor for redox reactions. The cysteine residues at its end undergo modifications through these reactions, causing a conformational change in Keap1. This change allows Nrf2 to dissociate and translocate into the nucleus, in which it forms a heterodimer with Maf protein^21^. The Nrf2-Maf complex then binds to the antioxidant response element (ARE) to activate the expression of target genes^2+^. These include phase II metabolic enzymes and antioxidant enzymes such as HO-1, SOD, peroxidase-1, glutathione (GSH), and γ-glutamylcysteine synthetase^23^. This activation helps to counteract oxidative damage and restore cellular homeostasis.

In this study, pretreatment with grape seed proanthocyanidin B2 significantly upregulated the expression levels of Nrf2 and HO-1 proteins in renal tubular epithelial cells induced by hypoxia-reoxygenation. However, the expression levels of Nrf2 and HO-1 were significantly downregulated upon addition of the Nrf2 inhibitor BRU. These findings suggested that grape seed proanthocyanidin B2 pretreatment activates the Nrf2/HO-1 signaling pathway, enhances the expression of Nrf2 and HO-1 proteins, and helps maintain the stability of the intracellular antioxidant system, thereby reducing oxidative stress damage to the cells.

The endoplasmic reticulum is a crucial organelle involved in the synthesis of proteins and lipids, as well as the folding, assembly, and transport of peptide chains. Under various stimuli such as ischemia, hypoxia, oxidative stress, acidosis, energy depletion, and calcium imbalance, cells can experience misfolding and aggregation of proteins in the endoplasmic reticulum lumen, leading to disruptions in endoplasmic reticulum function. This condition is known as endoplasmic reticulum stress^24^. When endoplasmic reticulum stress occurs, cells activate an unfolded protein response. This response triggers the expression of downstream target genes that help degrade unfolded or misfolded proteins, maintain endoplasmic reticulum homeostasis, and promote cell survival^25^. However, when endoplasmic reticulum stress is prolonged or severe, excessive activation of the unfolded protein response can lead to apoptosis^26^.

GRP78 is an endoplasmic reticulum chaperone heat shock protein that plays a crucial role in maintaining endoplasmic reticulum homeostasis and promoting protein folding^27^. CHOP is a transcription factor associated with apoptosis and plays a crucial role in diseases mediated by endoplasmic reticulum stress. Prolonged activation of the unfolded protein response can induce cell apoptosis, primarily through the activation of the PERK-eIF2α-ATF4-CHOP signaling pathway. When endoplasmic reticulum homeostasis is disrupted and endoplasmic reticulum stress occurs, it triggers the unfolded protein response. This response activates PERK, leading to its autophosphorylation. Phosphorylated PERK then phosphorylates eIF2α, which in turn increases the expression of the downstream protein ATF4. Elevated ATF4 further activates CHOP, and increased CHOP expression can subsequently induce cell apoptosis.

Zhang et al.^28^ established mouse models of renal ischemia-reperfusion injury and hypoxia-reoxygenation in HK-2 renal tubular epithelial cells. Their results showed that during renal ischemia-reperfusion injury and hypoxia-reoxygenation of renal tubular epithelial cells, the expression levels of endoplasmic reticulum stress-related proteins GRP78 and CHOP, and the apoptotic protein caspase-12 were significantly upregulated28. These findings indicated that endoplasmic reticulum stress and apoptosis induced by endoplasmic reticulum stress are present during renal ischemia-reperfusion injury. Additionally, previous studies have found that endoplasmic reticulum stress can activate the CHOP signaling pathway, which exacerbates apoptosis in renal tubular epithelial cells during ischemia-reperfusion injury^29^.

In this study, we established a hypoxia-reoxygenation model of renal tubular epithelial cells and applied pretreatment with grape seed proanthocyanidin B2. The results revealed that during hypoxia-reoxygenation, there was an upregulation of endoplasmic reticulum stress-related proteins GRP78 and CHOP, along with an increase in the expression of the apoptotic protein cleaved-caspase-3. Additionally, pretreatment with grape seed proanthocyanidin B2 significantly inhibited the expression of endoplasmic reticulum stress-related proteins GRP78 and CHOP, as well as the apoptotic protein cleaved-caspase-3, induced by hypoxia and reoxygenation. This treatment markedly reduced endoplasmic reticulum stress and apoptosis in renal tubular epithelial cells.

## Conclusion

The *in-vitro* study results indicated that grape seed proanthocyanidin B2 pretreatment significantly alleviates mitochondrial damage in HK-2 cells induced by hypoxia-reoxygenation, helps maintain the stability of mitochondrial membrane potential, and notably reduces calcium overload in HK-2 cells caused by hypoxia-reoxygenation. Furthermore, grape seed proanthocyanidin B2 pretreatment significantly upregulates the expression of antioxidant proteins Nrf2 and HO-1, while inhibiting the expression of endoplasmic reticulum stress-related proteins GRP78, CHOP, and the apoptotic protein cleaved-caspase3. This protective effect may be attributed to the ability of grape seed proanthocyanidin B2 pretreatment to significantly activate the Nrf2/HO-1 signaling pathway while inhibiting the CHOP signaling pathway. However, further *in-vivo* and animal studies are needed to confirm these experimental findings.

## Data Availability

The data will be available from the corresponding author upon reasonable request.
